# Retraction Note: Silencing of TRIM11 suppresses the tumorigenicity of chordoma cells through improving the activity of PHLPP1/AKT

**DOI:** 10.1186/s12935-023-03017-2

**Published:** 2023-09-26

**Authors:** Bin Wang, Gang Wang, Qingfeng Wang, Ziqiang Zhu, Yunqing Wang, Kangwu Chen, Huilin Yang

**Affiliations:** 1 Department of Orthopaedic Surgery, The First Affiliated Hospital of Soochow University, Shizi Rd 188, Suzhou, 215006 Jiangsu People’s Republic of China; 2 Department of Orthopaedic Surgery, The Second Affiliated Hospital of Xuzhou Medical University, Xuzhou, 221000 Jiangsu People’s Republic of China

**Retraction Note: Cancer Cell Int (2019) 19:284** 10.1186/s12935-019-1007-7

The Editors-in-Chief have retracted this article. Concerns were raised regarding a number of figures, specifically:

- Figure 2f appears to overlap with Figure 8B and 8D from [1].
- Figure 6d appears to overlap with Figure 6F from [2].
- Figure 4c appears to overlap with Figure 3C from [3].
- Figure 2D appears overlap with Figure 1F from [4].

The Editors-in-Chief therefore no longer have confidence in the results and conclusions this article.

The authors agree with this retraction.
